# Diagnostic delays, treatment timeliness, and patient experiences in triple-negative breast cancer: a mixed-methods study

**DOI:** 10.3389/fonc.2026.1826987

**Published:** 2026-08-04

**Authors:** Marcela Mazo Canola, Rebekkah Schear, Laura T. Housman, Kandis V. Backus, Cosmina Hogea

**Affiliations:** 1 Division of Hematology-Oncology, University of Texas Health San Antonio, San Antonio, TX, United States; 2 Polaris Global Health Solutions, Austin, TX, United States; 3 Avalere Health, Washington, DC, United States; 4 Gilead Sciences, Inc., Foster City, CA, United States

**Keywords:** cancer care pathways, care coordination, mixed-methods, patient experience, patient navigation, patient outcomes, time to treatment, triple-negative breast cancer

## Abstract

**Background:**

Triple-negative breast cancer (TNBC) is an aggressive breast cancer subtype that disproportionately affects younger patients, and Black and Hispanic/Latina patients. Timely diagnosis and treatment initiation are critical for optimal outcomes, yet variation in care delivery and delays in treatment initiation persist. While administrative data have documented differences in treatment timeliness, less is known about how patients experience the early TNBC care pathway and how these experiences relate to observed healthcare utilization patterns.

**Methods:**

We conducted a convergent parallel mixed-methods study integrating qualitative interviews with patients diagnosed with TNBC and a retrospective analysis of administrative claims data. Semi-structured interviews were conducted with 42 patients in the United States, intentionally oversampling Black, Hispanic/Latina, and younger patients. Quantitative analyses examined screening, diagnostic utilization, time to treatment initiation, and post-treatment care utilization among 6,345 commercially insured patients and patients with Medicaid managed care (MMC). Qualitative and quantitative findings were analyzed independently and then integrated to identify areas of convergence, divergence, and expansion.

**Results:**

Quantitative analyses identified observed differences in adjusted time to neoadjuvant treatment initiation among Black and Hispanic/Latina patients enrolled in MMC, with adjusted mean times increasing from 44.2 days (95% CI: 40.7–48.0) among White patients to 63.2 days (95% CI: 58.1–68.8) among Hispanic/Latina patients and 82.1 days (95% CI: 75.3–89.4) among Black patients. Qualitative findings provided context for these observed patterns, with participants describing symptom dismissal, difficulties translating complex clinical information into meaningful understanding, insurance-related barriers, and logistical challenges that may affect escalation and treatment readiness. Divergences highlighted limitations of claims-based measures, including their inability to capture emotional distress, uncertainty, feeling unseen within care interactions, fragmented care experiences, and inadequate preparation for treatment side effects.

**Conclusion:**

By integrating patient experiences with claims-based analyses, this study provides hypothesis-generating insights into early TNBC care pathways and identifies potential inflection points where health system interventions, particularly patient navigation and tailored education, may support more equitable, coordinated, and patient-centered care, across the TNBC care continuum.

## 1 Introduction

Triple-negative breast cancer (TNBC) is an aggressive subtype of breast cancer that accounts for approximately 15–20% of all breast cancers in the United States (1). TNBC disproportionately affects Black patients, Hispanic/Latina patients, and younger patients (2). Research has shown that these groups experience higher incidence and worse outcomes compared with White patients: key studies in the last decade have articulated that young patients with TNBC exhibit more aggressive tumor biology, with higher-grade tumors and higher proliferative index (Ki-67) compared to older patients (3–5). Similarly, Black and Hispanic/Latina patients experience lower five-year survival relative to White patients (6), and Black patients have the lowest stage-specific survival for TNBC of any racial or ethnic group (7). These findings underscore the importance of optimizing the TNBC care pathway and patient experience for younger patients, Black patients, and Hispanic/Latina patients, who may face elevated risks of delayed diagnosis, treatment barriers, and fragmented care.

TNBC lacks the three commonly targeted receptors: ER, PR, and HER2, thus historically limiting treatment to cytotoxic chemotherapy (8). Recently, immunotherapy has been shown to improve TNBC outcomes (9) when delivered promptly and in early stages (10). Because TNBC progresses quickly and has fewer therapeutic options, timely diagnosis and treatment initiation are essential (11); even modest delays can limit access to the most effective therapies, intensify symptom burden, and worsen quality of life. Yet timeliness alone does not define high-quality TNBC care. Treatment timing shapes some of the outcomes that research shows are most important to patients, including how they function and feel. But other key domains such as communication, coordination, symptom management, and supportive care also influence patients’ ability to understand their diagnosis, participate in decisions, tolerate treatment, and maintain well-being. Improving TNBC care requires a clear understanding of how patients move through detection and diagnosis, including factors contributing to early diagnostic delays, as well as understanding the broader patient experience of diagnosis, treatment preparation, early treatment, and supportive care. Identifying where care becomes difficult for patients is essential to addressing barriers that undermine treatment and overall well-being across the care continuum.

To our knowledge, this is the first TNBC-specific mixed-methods study to comprehensively characterize patient experiences across the early care pathway, with deliberate focus on Black, Hispanic/Latina, and younger patients. Existing literature is dominated by epidemiologic, registry-based, and molecular outcome studies, while qualitative or mixed-methods research on patient experience represents only a fraction of TNBC scholarship. Yet qualitative data provides essential insight into how patients understand their diagnosis, navigate complex treatments, engage in shared decision-making, and manage emotional, informational, and supportive-care needs. Incorporating these perspectives fills a knowledge gap that is crucial for developing patient-centered educational tools, communication strategies, and interventions that align with lived realities of patients.

This mixed-methods study explores the lived experience of the care pathway for patients with TNBC in the United States and integrates quantitative analyses of trends in treatment and utilization from claims data to generate a more comprehensive understanding of gaps, unmet needs, and opportunities to improve TNBC care. By situating quantitative patterns within the context of lived experiences of patients, this study aims to identify clinically relevant insights and impacts to enhance early detection, diagnostic timeliness, treatment coordination, high-quality care delivery, and whole-person support across the TNBC care continuum.

## 2 Materials and methods

### 2.1 Overview of study design

We conducted a convergent parallel mixed-methods study integrating qualitative interviews with 42 patients diagnosed with TNBC and a retrospective secondary analysis of administrative claims data from 6,345 patients. Quantitative and qualitative data were analyzed independently and then integrated and analyzed to identify:

- Areas of convergence: areas where qualitative narratives aligned with quantitative patterns
- Areas of divergence: areas where qualitative narratives did not align with quantitative patterns.
- Additional dimensions: barriers or opportunities revealed only through qualitative interviews.

**Figure 1:**
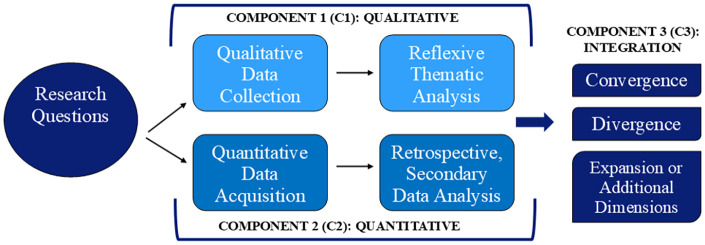
Overview of mixed-methods convergent parallel design. Adapted from Chicoine et al., (12) BMJ Open 2021, used under the terms of the Creative Commons Attribution (CC BY 4.0) license.

**Figure 1** presents an overview of the convergent parallel design, with subsequent sections providing detailed descriptions of qualitative and quantitative components.

### 2.2 Qualitative component

The qualitative component (Component 1, or C1) used a purposive quota-based sample of patients diagnosed with TNBC and receiving pharmacologic treatment between July 1, 2017, and June 30, 2022 (n=42). Participants were recruited through a third-party recruitment vendor using established patient panels and underwent eligibility screening and demographic assessment prior to enrollment. Eligibility criteria included a self-reported TNBC diagnosis, receipt of pharmacologic treatment during the study period, access to an internet-connected device with audio capability, ability to read English, and provision of verbal informed consent. Recruitment targets were established *a priori* to approximate incidence patterns among White and Black patients while intentionally oversampling Hispanic/Latina patients due to their lower representation in the broader study population; however, final enrollment reflected both recruitment feasibility and thematic saturation. The final sample included 17 Black patients, 8 Hispanic/Latina patients, 10 White patients, and 7 non-white patients of other racial backgrounds (Asian, American Indian, or multiracial patients). Potential participants completed IRB-approved informed consent, screening, and demographic questionnaires to assess eligibility. Interviews were conducted by four trained research team members who were matched to patients based on shared racial and ethnic identities, when feasible. The study was approved by the Institutional Review Board Advarra (Columbia, MD). The discussion guide supported a semi-structured in-depth interview, with a mix of structured questions and open-ended questions that allowed interviewees to share experiences authentically in their own words. Interview questions focused on the following domains:

- Experience with screening, diagnostic procedures, and cancer diagnosis.
- Timeline of diagnostics, referrals, receipt of results, and treatment.
- Communication about diagnosis.
- Preparation, information, and education for treatment, symptoms, and side effects.
- Readiness for treatment.
- Treatment experience.
- Provision of supportive resources and care.
- Gaps in care and examples of high-quality care.
- Experiences with unfair treatment due to race, cultural background, or type of insurance coverage

Recruitment and interview progression were discussed during weekly meetings among the four researchers who conducted interviews, and the interview team determined that sufficient depth and breadth of perspectives had been achieved following completion of all 42 interviews. Interview transcripts were cleaned and deidentified by the initial interviewing team. Transcripts were imported into DelveTool (13) for qualitative coding and theme development by the corresponding author using Braun and Clarke’s reflexive thematic analysis approach (14–16). She used an iterative and interpretive process focused on identifying patterns of shared meaning across participant experiences. Coding was conducted inductively, with descriptive and interpretive codes generated and iteratively refined through repeated engagement with the data. The analysis was informed by an experiential qualitative orientation focused on understanding how participants described and interpreted their experiences navigating TNBC diagnosis and treatment, while also incorporating a patient-centered health services research perspective, acknowledging the role of researcher interpretation in theme development. Specifically, the researcher’s experience in oncology and patient-centered research allowed her to move recursively between transcripts, emerging patterns, and relevant literature to refine conceptual understanding, deepen interpretation, and generate themes (17). Throughout the analysis, consideration was given to how prior experiences and assumptions could shape the interpretation of participant narratives, and transcripts were iteratively reviewed to support the grounding of themes in participant accounts. Co-authors reviewed and provided feedback on thematic organization and interpretation during manuscript development.

### 2.3 Quantitative component

Component 2 (C2) was comprised of a quantitative analysis, whereby researchers conducted a retrospective analysis of claims data from Medicaid managed care (MMC) and commercial health plans through Inovalon’s MORE^2^ (Medical Outcomes Research for Effectiveness and Economics) Registry^®^, a multi-payer database that captures the enrollment and claims data of more than 338 million commercial, Medicare FFS, Medicare Advantage, and Managed Medicaid beneficiaries, as well as Acxiom’s U.S.-based InfoBase^®^ Geo database (Conway, AR), to integrate patient-specific social determinants of health information at the 5-digit ZIP code level (18). The quantitative study population included adult patients with TNBC enrolled in commercial (n=4,494) and MMC (n=1,851) healthcare plans between January 1, 2017, and June 30, 2021. At the time of analysis (2023) and during the study period evaluated (2017–2021), no TNBC-specific ICD-10-CM code was available; therefore, direct subtype identification through administrative coding was not possible. Claims-based approaches have previously been used to identify breast cancer events when direct clinical information is unavailable, and administrative algorithms integrating diagnoses, procedures, and treatment information have demonstrated utility for identifying breast cancer-related outcomes (19, 20). Similar treatment- and diagnosis-based proxy approaches have also been applied in TNBC and other breast cancer populations when direct subtype information is unavailable (21, 22). Therefore, TNBC was identified using a claims-based algorithm requiring evidence of breast cancer diagnosis (≥1 inpatient claim or ≥2 outpatient claims, with the first qualifying claim serving as the index date), absence of hormone receptor-positive (HR+) or HER2-positive therapy claims during the 12 months following index, and ≥1 TNBC-related treatment claim during the same period. Continuous medical and pharmacy enrollment for ≥12 months before the index was required, along with no evidence of other neoplasms during the specified study windows. Although TNBC-specific ICD-10-CM codes have since become available, these were not available during the study period and therefore could not be applied in the present analysis.

Patients were categorized into treatment cohorts according to the timing of systemic therapy relative to surgery. Neoadjuvant treatment was defined as systemic therapy initiated within 45 days before surgery, while adjuvant treatment was defined as systemic therapy initiated within 45 days following surgery. Patients whose systemic therapy occurred outside these windows were classified as advanced/metastatic. The presence of metastatic diagnosis codes was used as a confirmatory indicator for advanced/metastatic classification, but was not required, as metastatic coding may be inconsistently captured in administrative claims because it is not uniformly required for reimbursement. Because the analysis focused on pharmacologic treatment patterns, inclusion required evidence of qualifying systemic drug therapy; therefore, patients undergoing surgery solely for palliative purposes without qualifying systemic treatment would not have met analytic inclusion criteria. Clinical characteristics and treatment utilization were pulled directly from medical and pharmaceutical claims data. Member-level data, such as age, region, and rural/urban residency, were obtained from enrollment files and linked with social determinants of health (SDOH) characteristics from Acxiom’s InfoBase^®^ Geo files. SDOH data were linked to the member’s 9-digit ZIP Code, which reflects approximately 30 million “near neighborhood” areas representing an average of five households, and has been used as a more granular proxy approach than larger geographic units in health services research, for person-level SDOH characteristics (23). Race and ethnicity were assigned using self-reported enrollment information when available. When race/ethnicity information was unavailable, classifications were imputed using Acxiom near-neighborhood estimates linked at the 9-digit ZIP Code level. Race/ethnicity data were missing for 21% of the Medicaid managed care cohort and 85% of the commercial cohort. Imputed race/ethnicity values were used in analyses involving race-stratified comparisons and multivariable analyses including race/ethnicity as an independent variable. Distribution of race/ethnicity assignments following imputation was similar to distributions observed in enrollment data and publicly available estimates. Domains of interest included combinations of treatments, timeliness of initiation of treatment, and treatment utilization. Univariate analyses were used to describe sample characteristics, and multivariable analyses examined associations between patient characteristics and time-to-treatment outcomes. Time-to-event analyses were conducted using generalized linear models implemented with PROC GENMOD in SAS version 9.4, specifying a Poisson distribution with a log link function. Model estimates were generated on the log scale, and exponentiated estimates (ExpEstimate values) were reported to facilitate interpretation of model results on the original scale. Least squares means (LS means) were used to estimate adjusted effects for covariate patterns represented within the model. Covariates included rurality (urban vs rural), race/ethnicity (White, Black, Hispanic/Latina), metastatic status (C77–C79 diagnosis codes within 12 months post-index), census region, Health Professional Shortage Area (HPSA) designation, age group (<30, 31–40, 41–50, 51–64, 65–74, ≥75 years), Charlson Comorbidity Index score (0 vs >0), household income category (low, medium, high), and prior screening or diagnostic procedures including mammography, ultrasonography, and digital tomosynthesis. Covariates were selected using a combination of empirical findings from descriptive analyses and variables considered clinically or conceptually relevant to the study objectives. Interaction terms were not included in the prespecified analytic approach. Missing values for variables other than race/ethnicity were categorized as “Missing/Other,” where applicable. Race and ethnicity were assigned using observed enrollment data when available and imputed using Acxiom near-neighborhood estimates when missing, as described above. Sensitivity analyses restricted to observed race/ethnicity data were not conducted. Researchers analyzed demographics, screening and diagnostic testing, patient treatment experience, and healthcare utilization. Results were stratified by Acxiom’s near neighborhood ZIP code linkages as to whether patients lived in rural vs non-rural areas and whether they were identified as White, Hispanic/Latina, Black, or “Missing or Other” race or ethnicity, and by insurance type. Key definitions included:

- Time to treatment: days between TNBC diagnosis and the first of either surgery or chemotherapy.
- Adjuvant treatment: patients had a relevant surgical procedure 45 days or more before chemotherapy.
- Neoadjuvant Treatment: patients had a relevant surgical procedure 45 days or more after chemotherapy.
- Advanced or metastatic treatment: patients who did not have a relevant surgical procedure within a 45-day window of chemotherapy.

The metastatic flag was assigned based on the presence of a secondary ICD-10 diagnosis code for metastasis and was used for exploratory sub-analyses by the independent variables noted above. Rural areas were defined as a small town or rural area; urban areas were defined as metropolitan or micropolitan areas, based on rural-urban commuting area codes.

## 3 Results

### 3.1 Qualitative findings

Qualitative interviews illuminated the lived experiences of patients navigating TNBC diagnosis and treatment and identified nine interconnected themes describing experiences across the care pathway. Themes reflected symptoms not matching patient and provider expectations resulting in delayed recognition; diagnosis- and care-related communication challenges; feeling unseen in healthcare interactions; administrative systems creating obstacles to care navigation; fragmented care experiences; challenges interpreting symptoms; patient unfamiliarity with medical information; ongoing emotional burden and uncertainty; disruptions to daily roles and responsibilities; and unexpected treatment experiences that left patients feeling underprepared to cope physically or emotionally.

Together, these themes reflected how interpersonal, structural, emotional, and practical factors shape care-seeking behavior, access to timely diagnosis and treatment, engagement with shared decision-making, experiences of care, and quality of life.

**Table 1** summarizes these themes, primary insights, potential impacts on care pathways, and representative quotes.

**Table 1:** Summary of qualitative themes, key insights, impacts, and quotes.

Theme	Primary insight	Potential impact on care pathways	Representative quotes
Theme 1: Experiences of dismissal and delayed recognition	Participants described symptoms through expectations around age, breastfeeding, postpartum experiences, and perceived cancer risk, contributing to delayed recognition and help-seeking.	Under-recognition of symptoms by patients and providers may contribute to delayed evaluation and lower urgency in diagnostic workup	*“I call my general practitioner, and I heard this a lot during the diagnosis point and after, ‘Well, you’re so young, you shouldn’t have breast cancer.’ And so that delayed [tests], and the urgency of everything.”* *“She was certain that it was stage 4, and she told me to quit my job, cash out my 401K, and enjoy my kids because she gave me two years [to live]. And this was just for nothing, because it turns out it wasn’t [stage four].”* *“I had a young and healthy lifestyle and didn’t smoke. I know we had to do mammograms when we were turning 50. {Now], I know it’s 40. I was just waiting, but it caught up to me.”* *“I’m thinking, ‘Well, it’s close to my period. Maybe it’s just a lump having to do with that.’ So, I kind of dismissed [my symptom] for a little while.”*
Theme 2: Diagnosis- and care-related communication gaps and information overload	Participants described difficulty translating complex clinical information into meaningful understanding during often emotionally overwhelming periods.	Increased emotional distress and reduced capacity to engage in shared decision-making	*“[His poor] communication was one of the reasons that I switched oncologists.”* *“It was the first time I ever heard the term triple negative. No one I knew had ever heard of it.”* *“To them it’s just another procedure, but for me it was life-changing.”*
Theme 3: Feeling unseen: identity, representation, and trust in care	Participants described experiences where age, race/ethnicity, gender, or life stage influenced whether they felt understood and represented in care interactions.	Trust being dependent on feeling ‘seen’ by a provider; lack of feeling ‘seen,’ resulting in hesitation to ask questions, and feeling isolated	*“I’m a Black woman under 40. How are you [my oncologist] connecting with me?”* *“Being a young person with cancer, there weren’t a lot of other young people going through treatment in the same clinic as me.”* *“I switched oncologists to a bigger city nearby, a female oncologist [who] has a better understanding of me, and [of] my thoughts, and explains things better.”*
Theme 4: Administrative systems as obstacles to care navigation	Participants described insurance and administrative requirements as barriers requiring additional effort, persistence, and self-advocacy.	Delayed diagnostics and treatment initiation with emotional and financial strain	*“If you did not have the medical coverage that fit what they [health insurance] were trying to get, it was like, ‘Oh well, we’ll just do our best.’ As a nation, we’re very callous if your medical coverage doesn’t cover [a diagnosis]. It’s just: ‘Oh well.’”* *“[My oncologist] had to speak with the insurance four times. Why does the insurance make it so difficult? It was only after he jumped through these hoops [that] they approved the PET scan. [By then], I had metastasis to my brain.”*
Theme 5: Fragmented care and poor coordination	Participants described disconnected care processes and limited access to specialized services, particularly in geographically constrained settings.	Missed follow-up steps and reduced access to treatment opportunities	*“They don’t offer clinical trials in this town … I didn’t realize that until this came up.”* “*I had another good friend tell me, ‘I know how you feel because I’ve had so many members of my family have cancer,’; to which I replied, ‘No, you don’t know how I feel until it’s happened to your body.’”*
Theme 6: Challenges in interpreting symptoms and navigating unfamiliar medical information	Participants described uncertainty when attempting to interpret symptoms and navigate unfamiliar terminology and care processes.	Misunderstanding and disengagement from care	*“I dismissed it, thinking it was hormonal.”* “*I noticed a lump, and I was thinking it was clogged milk ducts, and that it would eventually turn into mastitis. I tried massaging, and nothing ever worked out. It would have been April before I finally went to the doctor.”*
Theme 7: Living with uncertainty, distress, and emotional burden	Participants described ongoing fear, uncertainty, emotional distress, and feelings of isolation that extended beyond diagnosis into survivorship.	Mental health strain may affect engagement with care and quality of life	*“I don’t remember what else she said because I blanked out completely. I hung up the phone and immediately started sobbing. I wailed. I just lost it, because you think the worst.”* *“And then after that, it’s like, ‘Alright, is [the cancer] gonna come back?’”* *“I felt very alone in a rural area.”*
Theme 8: Disruptions to daily life, roles, and responsibilities	Participants described substantial disruptions to day-to-day functioning in work, caregiving, transportation, and maintaining daily responsibilities.	Practical burdens may complicate care participation and increase financial strain.	*“There were appointments on top of appointments: scans, blood work, specialists, and it felt like my entire life revolved around trying to get to the next one. It was hard to keep up with work and everything else in my life at the same time.”* *“I couldn’t see, so I couldn’t drive anymore. Someone had to take me to every appointment. That was [a] side effect that really changed my day-to-day life.”* *“It’s difficult when you work business hours, and the clinics are only open business hours. My follow-ups are supposed to be every six months, and I’m coming up on nine [months] because it’s hard to make scheduling work.”*
Theme 9: Unexpected treatment experiences and feeling underprepared	Participants described feeling inadequately prepared for the physical and emotional consequences of treatment.	Limited anticipatory guidance may worsen distress and treatment experiences.	*“The treatment was explained well, but the drugs that counteract the effects of the chemo weren’t explained much. I was just in a hurry to get done, but I think part of [my being underprepared for treatment] is my fault as well. It wasn’t explained clearly.”* *“I didn’t know what [treatment] would pose on my mental health. I knew about the physical side effects, but I wasn’t prepared for the anxiety, the depression, or how long those things would last after treatment ended.”*

Through interviews, patients described symptoms being interpreted through *their or their providers’* expectations related to age, postpartum experiences, and perceived cancer risk, contributing to delayed recognition and care-seeking. Participants also described communication experiences that heightened confusion and uncertainty, feeling unseen or misunderstood within healthcare interactions, insurance and administrative barriers requiring substantial self-advocacy, fragmented care experiences, and significant disruptions to daily functioning and social roles during diagnosis and treatment.

A pre-menopausal patient was diagnosed only after unsuccessfully trying to convince her gynecologist to send her for a diagnostic ultrasound, and had to self-advocate for a biopsy:

“I found a tiny, tiny lump. I called the gynecologist. She did this touch exam; she could not detect it. She said, ‘Oh, I don’t feel anything, you know it could be [that you should] just stop breastfeeding.’ And I said, ‘Well, I wanna check this because [the lump] wasn’t there [before].’ She sent me [for] a normal mammogram. I said, ‘I still [want a] mammogram because this wasn’t there.’ She didn’t schedule a diagnostic mammogram, so I had to call her. I said, I wanna diagnostic, and she said, Well, we’ll just watch the mass.’ When I went for the mammogram, they said, ‘We can’t tell it apart, so we can wait six months and check again, or we can biopsy.’ I wanted the biopsy; it turns out that it was invasive ductal carcinoma.”

Another pre-menopausal patient’s own lack of knowledge about signs and symptoms, misinformation, and belief about her risk based on her younger age contributed to her delayed diagnosis:

“I was just really stupid in thinking that it was nothing, because, honestly, I have super small breasts, and I’m like, ‘well, at least I never have to worry about getting breast cancer.’ And I would say that out loud to my family…. Like, to my mom. Just because you think, well, you would know it right away if you had it, because you’d be able to find it. It’s not hidden in these large boobs.’ So, I always thought that was something that could never happen to me.”

A patient living in a rural setting experienced a lack of geographic access to high-quality, specialized care because of her rural geography:

“I didn’t feel locally that they really knew enough about the diagnosis, but felt like they need to know a lot more.”

Another patient mentioned insurance and financial issues impacting her access to high-quality care locally, causing her to seek care internationally:

“The insurance price made my post-treatment procedures impossible in this country. That’s the conclusion. I had to seek treatment abroad.”

A patient discussed her experience where medical errors caused delays in scheduling her treatment, and her resulting confusion and frustration:

“They did my lung biopsy. It came back as inflammation in my lungs, that’s what they saw. So, it wasn’t [metastatic] cancer [like I had been told]. And then we canceled the clinical trial [they had set me up on] and rescheduled my surgery [again].”

A Black patient shared her preference for a provider who understands her needs and identity:

“What would have been helpful for me was the more personalized approach to [my care]. Meaning, [if] my doctor[could] make it a point to state that he works with a lot of patients of color; how he’s done research, and he has done his homework to make sure that I know that he is aware of the challenges and barriers that are in the system for me.”

Overall, these themes illuminate multilevel barriers that influence patients’ diagnostic journeys, treatment readiness, and experiences of care, along with the practical aspects of their day-to-day lives; factors that quantitative measures alone cannot fully capture.

### 3.2 Quantitative findings

#### 3.2.1 Sample characteristics

**Table 2** summarizes demographic and geographic characteristics of the study population stratified by insurance type, race and ethnicity, and age.

**Table 2:** Distribution of age, race/ethnicity, and geography among commercially insured and MMC patients (N = 6,345).

Commercial (N = 4,494)			Medicaid managed care (N = 1,851)		
	Frequency	Percentage of total		Frequency	Percentage of total
**Age**			**Age**		
<30	56	1.2%	<30	55	3.0%
31-40	451	10.0%	31-40	296	16.0%
41-50	1,106	24.6%	41-50	485	26.2%
51-64	2,308	51.4%	51-64	779	42.1%
65-74	472	10.5%	65-74	173	9.3%
75+	101	2.2%	75+	63	3.4%
**Race/Ethnicity**			**Race/Ethnicity**		
White	2892	64.4%	White	750	40.5%
Black or African American	770	17.1%	Black or African American	637	34.4%
Hispanic or Latina	428	9.5%	Hispanic or Latina	330	17.8%
Asian	175	3.9%	Asian	105	5.7%
Unknown/Missing	229	5.1%	Unknown/Missing	29	1.6%
**Geography**			**Geography**		
Rural	262	5.8%	Rural	96	5.2%
Not Rural	3949	87.9%	Not Rural	1,573	85.0%

Figure data source: MORE^2^ Registry for 2019–2021.

Across both insurance groups, most patients were between the ages 41 and 64. Racial and ethnic composition differed substantially by insurance category: the commercial cohort was predominantly White (64.4%), while the MMC cohort included a higher proportion of Black (34.4%) and Hispanic/Latina (17.8%) patients. Rural residence was uncommon in both cohorts but proportionally similar (5.8%: Commercial and 5.2%: MMC). These distributions provide important context for understanding differences in treatment pathways and timeliness observed in subsequent analyses.

#### 3.2.2 Utilization of screening, diagnostic services, and telemedicine

To contextualize patterns of care utilization before and after TNBC diagnosis, we examined utilization of breast cancer screening, diagnostic imaging, biopsy procedures, genetic counseling, and narrow genetic testing (BRCA testing specifically), as well as telemedicine use across geographic residence and race/ethnicity. **Table 3** shares a summary of the results. Note that some estimates are suppressed in tabular presentation (values represented by “--”) due to low cell counts, absent records, or data use/privacy requirements, and do not indicate missing data. Percentages represent the proportion of patients within each corresponding subgroup (e.g., rural, non-rural, White, Hispanic/Latina, Black patients). Finally, statistically significant comparisons highlighted in the Results text are indicated with corresponding p-values where applicable.

**Table 3:** Utilization of screening, diagnostic, and telemedicine services by rurality, race/ethnicity, and payer.

COMMERCIAL POPULATION								
n = 4,494								
Variable	Rural n = 262 (6%)	Non-rural n = 3,949 (88%)	p-value (Rural vs Non-rural)	White, Non-Hispanic n = 2,892 (64%)	Hispanic/Latina n = 428 (10%)	p-value (Hispanic/Latina vs White)	Black n = 770 (17%)	p-value (Black vs White)
Mammograms in 12 months pre-index	104 (40%)	1,578 (40%)	0.9325	1,144 (40%)	162 (38%)	0.4999	320 (42%)	0.3138
Ultrasonography in 12 months pre-index	217 (83%)	3,218 (81%)	0.5893	2,363 (82%)	342 (80%)	0.3706	628 (82%)	0.9240
Digital tomosyntesis in 12 months pre-index	146 (56%)	2,604 (66%)	0.0008	1,963 (68%)	238 (56%)	0.0001	494 (64%)	0.0508
Diagnostic MRI in 3 months pre- and 6 months post-index	72 (27%)	1,335 (34%)	0.0356	944 (33%)	150 (35%)	0.3232	278 (36%)	0.0702
Diagnostic mammogram in 3 months pre- and 6 months post-index	227 (87%)	3,468 (88%)	0.5732	2,535 (88%)	366 (86%)	0.2131	693 (90%)	0.0737
Tomosynthesis biopsy in 3 months pre- and 6 months post-index	<11 (--)	83 (2%)	--	52 (2%)	<11 (--)	--	27 (4%)	0.0037
Stereotactic-guided biopsy	12 (5%)	314 (8%)	0.0480	226 (8%)	27 (6%)	0.2730	56 (7%)	0.6162
MRI-guided biopsy	<11 (--)	264 (7%)	--	170 (6%)	31 (7%)	0.2692	58 (8%)	0.0914
Ultrasound-guided biopsy	211 (81%)	3,114 (79%)	0.5185	2,285 (79%)	337 (79%)	0.8972	615 (80%)	0.6018
Biopsy (nonspecific to modality)	13 (5%)	92 (2%)	0.0081	66 (2%)	18 (4%)	0.0180	18 (2%)	0.9272
Genetic counseling 12 months post-index	27 (10%)	534 (14%)	0.1378	396 (14%)	33 (8%)	0.0006	108 (14%)	0.8116
Narrow genetic testing: BRCA genetic tests 12 months post-index	48 (18%)	847 (21%)	0.2307	620 (21%)	86 (20%)	0.5256	161 (21%)	0.7500
Oncologist follow-up visits 3 months pre- and 12 months post-index	59 (23%)	647 (16%)	0.0100	554 (19%)	40 (9%)	0.0001	112 (15%)	0.0032
Telemedicine	46 (18%)	1,014 (26%)	0.0034	697 (24%)	136 (32%)	0.0006	208 (27%)	0.096
MEDICAID MANAGED CARE (MMC) POPULATION								
n = 1,851								
Variable	Rural n = 96 (5%)	Non-rural n = 1,573 (85%)	p-value (Rural vs Non-rural)	White, Non-Hispanic/Latina n = 750 (41%)	Hispanic/Latina n = 330 (18%)	p-value (Hispanic vs White)	Black n = 637 (34%)	p-value (Black vs White)
Mammograms in 12 months pre-index	24 (25%)	442 (28%)	0.5111	182 (24%)	95 (29%)	0.1170	200 (31%)	0.0031
Ultrasonography in 12 months pre-index	85 (89%)	1,374 (87%)	0.7323	654 (87%)	290 (88%)	0.7568	559 (88%)	0.7558
Digital tomosyntesis in 12 months pre-index	50 (52%)	861 (55%)	0.6123	462 (62%)	148 (45%)	0.0001	352 (55%)	0.0168
Diagnostic MRI in 3 months pre- and 6 months post-index	25 (26%)	531 (34%)	0.1195	268 (36%)	116 (35%)	0.8540	204 (32%)	0.1464
Diagnostic mammogram in 3 months pre- and 6 months post-index	77 (80%)	1,318 (84%)	0.3579	646 (86%)	264 (80%)	0.0108	534 (84%)	0.2304
Tomosynthesis biopsy in3 months pre- and 6 months post-index	<11 (--)	21 (1%)	--	<11 (--)	<11 (--)	--	<11 (--)	--
Stereotactic-guided biopsy	<11 (--)	98 (6%)	--	59 (8%)	19 (6%)	0.2174	28 (4%)	0.0079
MRI-guided biopsy	<11 (--)	79 (5%)	--	32 (4%)	21 (6%)	0.1417	35 (5%)	0.2879
Ultrasound-guided biopsy	79 (82%)	1,322 (84%)	0.6500	633 (84%)	272 (82%)	0.4170	534 (84%)	0.7723
Biopsy (nonspecific to modality)	<11 (--)	35 (2%)	--	15 (2%)	<11 (--)	--	23 (4%)	0.0671
Genetic counseling 12 months post-index	<11 (--)	135 (9%)	--	55 (7%)	24 (7%)	0.9719	57 (9%)	0.2713
Narrow genetic testing: BRCA genetic tests 12 months post index	<11 (--)	138 (9%)	--	65 (9%)	30 (9%)	0.8206	53 (8%)	0.8177
Telemedicine	33 (34%)	555 (35%)	0.8565	257 (34%)	141 (43%)	0.0079	210 (33%)	0.6098

Percentages represent the proportion of patients within each corresponding subgroup (e.g., rural, non-rural, White, Hispanic/Latina, Black/African American).

Values represented by “--” indicate suppressed estimates or p-values due to low cell counts, absent records, or data use/privacy requirements and do not indicate missing data.

Statistically significant comparisons highlighted in the Results text are indicated with corresponding p-values where applicable.

##### 3.2.2.1 Rural vs. non-rural residence variance

###### 3.2.2.1.1 In the commercial population

Compared with patients living in non-rural areas, those residing in rural areas had lower utilization of several advanced screening and diagnostic modalities. Rural patients were less likely to receive digital tomosynthesis in the 12 months before index diagnosis (56% vs. 66%, p=0.008), diagnostic breast MRI in the 3 months pre- to 6 months post-index (27% vs. 34%, p=0.036), and stereotactic biopsies within the same diagnostic window (5% vs. 8%, p=0.048).

###### 3.2.2.1.2 In the MMC population

Analyses restricted to Medicaid beneficiaries and residence strata did not yield statistically significant differences, likely due to limited sample size.

##### 3.2.2.2 Racial variance

###### 3.2.2.2.1 In the commercial population

Compared with White patients, Hispanic/Latina patients demonstrated lower utilization of digital tomosynthesis in the 12 months pre-index (56% vs. 68%, p=0.001), and of genetic counseling in the 12 months post-index (8% vs. 14%, p=0.006). Hispanic/Latina patients demonstrated lower oncology initial visit and follow-up visit utilization compared with White patients (9% vs 19%, p=0.0001). Similarly, Black patients showed lower patterns in initial and follow-up visits compared to White patients (15% vs 19%, p=0.003).

###### 3.2.2.2.2 In the MMC population

Compared to White patients, Hispanic/Latina patients demonstrated lower utilization of digital tomosynthesis (45% vs. 62%, p=0.001) and diagnostic mammography in the 3 months pre- to 6 months post-index (80% vs. 86%, p=0.01). Compared to White patients, Black patients had higher utilization of screening mammography in the 12 months pre-index (31% vs. 24%, p=0.003), but lower use of digital tomosynthesis (55% vs. 62%, p=0.017), stereotactic-guided biopsy (4% vs. 8%, p=0.008), and narrow genetic testing (88% vs. 91%, p=0.045). Among commercially insured patients, Black patients demonstrated trends toward higher diagnostic MRI (36% vs. 33%, p=0.07) and diagnostic mammography (90% vs. 88%, p=0.07) utilization, although these differences did not reach statistical significance.

###### 3.2.2.2.3 In commercial and MMC populations

Hispanic/Latina patients demonstrated higher telemedicine utilization compared to White patients:

- in the commercial population: (32% vs 24%, p=0.006)
- in the MMC population: (43% vs 34%, p=0.0079).

#### 3.2.3 Examining variance in time to treatment initiation among racial subgroups

**Figure 2:**
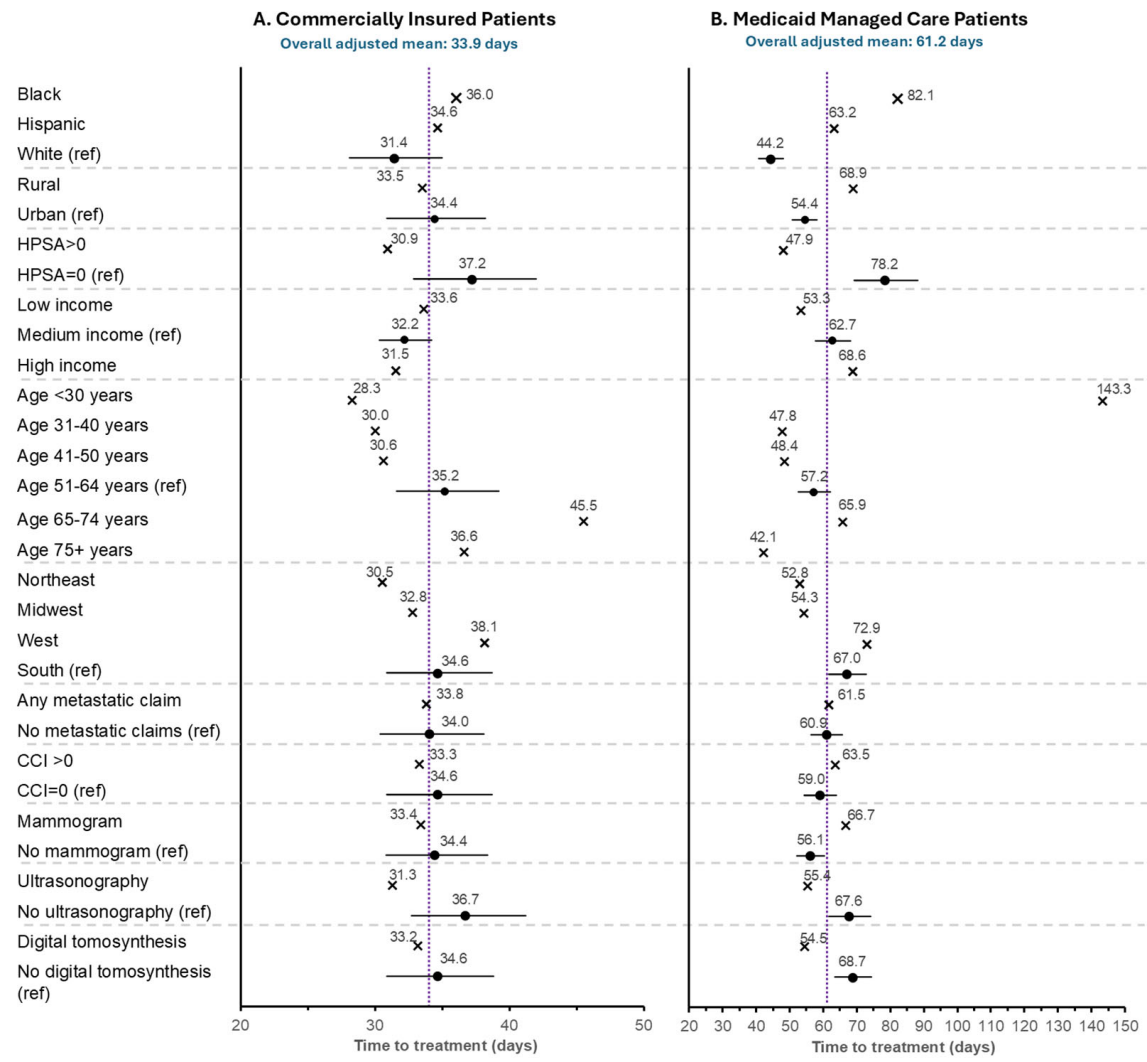
Multivariable covariate-adjusted estimates of time to neoadjuvant treatment initiation among **(A)** commercially insured and **(B)** Medicaid managed care patients.

To contextualize variation in treatment experiences, we examined model-adjusted time from diagnosis to treatment initiation across insurance categories. Patterns of delay differed by race and ethnicity across both insurance groups, although the magnitude of differences was most pronounced among MMC beneficiaries. **Figure 2** illustrates these covariate- adjusted estimates and 95% confidence intervals of time to neoadjuvant treatment initiation for both MMC and commercial groups, demonstrating the consistency and magnitude of differences across groups.

##### 3.2.3.1 In the MMC population

The adjusted time to neoadjuvant initiation ranged from approximately 44.2 days for White patients to 63.2 days for Hispanic/Latina patients and 82.1 days for Black patients, indicating significant differences and potentially clinically meaningful disparities.

##### 3.2.3.2 In the commercial population

Delays were also observed for Black and Hispanic/Latina patients compared with White patients, although the absolute differences were smaller (approximately 31–36 days overall), suggesting that differences associated with insurance type may contribute to variation in the magnitude of observed delays.

**Table 4** below shows the corresponding multivariable model estimates, including log-transformed coefficients (“Estimate”) and the back-transformed adjusted days to treatment (“ExpEstimate”). The ExpEstimate values represent the interpretable time to treatment in days and align with the patterns seen in the forest plot in **Figure 2**.

**Table 4:** Comparison of adjusted treatment timeliness across insurance types and racial or ethnic groups.

Insurance type and race/ethnicity	Estimate	StdErr	Z Value	P Value	Alpha	Lower	Upper	ExpEstimate	LowerExp	UpperExp
Medicaid – Neoadjuvant: Black	4.4077	0.04385	100.51	<.0001	0.05	4.3218	4.4937	82.0812	75.3209	89.4483
Medicaid – Neoadjuvant: Hispanic	4.1463	0.04319	96	<.0001	0.05	4.0617	4.4231	63.2023	58.0721	68.7857
Medicaid – Neoadjuvant: White	3.7883	0.0418	90.63	<.0001	0.05	3.7064	3.8703	44.1823	40.7068	47.9546
Medicaid – Adjuvant: Black	3.7292	0.03308	112.73	<.0001	0.05	3.6644	3.7941	41.6478	39.0331	44.4376
Medicaid – Adjuvant: Hispanic	3.7341	0.03381	110.46	<.0001	0.05	3.6678	3.8004	41.85	39.167	44.7169
Medicaid – Adjuvant: White	3.7038	0.03026	122.42	<.0001	0.05	3.6445	3.7631	40.601	38.2634	43.0814
Commercial – Neoadjuvant: Black	3.5824	0.05967	60.04	<.0001	0.05	3.4655	3.6994	35.9603	31.9912	40.4219
Commercial – Neoadjuvant: Hispanic	3.5436	0.06023	58.83	<.0001	0.05	3.4256	3.6617	34.5922	30.7404	38.9268
Commercial – Neoadjuvant: White	3.4455	0.0562	61.31	<.0001	0.05	3.3353	3.5556	31.3578	28.0874	35.009
Commercial – Adjuvant: Black	3.7507	0.0452	82.97	<.0001	0.05	3.6621	3.8393	42.5507	38.9429	46.4926
Commercial – Adjuvant: Hispanic	3.7687	0.04585	82.19	<.0001	0.05	3.6789	3.8586	43.3255	39.6015	47.3996
Commercial – Adjuvant: White	3.673	0.0438	83.86	<.0001	0.05	3.5871	3.7588	39.3679	36.1294	42.8967

In contrast to neoadjuvant treatment initiation, fully adjusted models revealed minimal racial or ethnic differences in time to adjuvant or advanced/metastatic treatment. Although unadjusted analyses indicated longer median delays for Black and Hispanic/Latina patients in several treatment settings, these differences appear attenuated or weakened in multivariable analyses, given that confidence intervals overlap. For example, in MMC, adjusted adjuvant initiation times were similar across groups: Black patients: 41.6 days (95% CI: 39.0–44.4), Hispanic/Latina patients: 41.9 days (39.2–44.7), and White patients: 40.6 days (38.3–43.1), these overlapping confidence intervals indicate that the estimated treatment times for Black, Hispanic/Latina, and White patients are not statistically different from one another once we adjust for all covariates.

Rural residence was not associated with delays in any multivariable model. In unadjusted analyses, rural patients initiated certain treatments earlier than non-rural patients, including earlier initiation of post-surgical advanced/metastatic and earlier adjuvant treatment among patients who did not receive neoadjuvant therapy. These patterns persisted after adjustment and were consistent across insurance groups, suggesting that rural residence was not likely a barrier to timely treatment in this dataset. Although adjusted differences may appear modest in some contexts (3–5 days in the commercial population), prior TNBC research demonstrates that even short delays can disrupt staging accuracy, narrow windows for initiating systemic therapy, and adversely affect survival (24).

### 3.3 Mixed-methods integration: convergence, divergence, and expansion of quantitative and qualitative findings

The mixed-methods integration used a convergent parallel design in which quantitative and qualitative data streams were analyzed independently and subsequently integrated through comparison of findings using a joint display approach (25). Integration focused on identifying areas of convergence (agreement), divergence (difference), and expansion (additional understanding generated by one data source beyond the other), allowing development of meta-inferences across datasets.

#### 3.3.1 Convergence

Quantitative analyses demonstrated that variances in timeliness of TNBC care were concentrated in the initiation of neoadjuvant therapy, with Black and Hispanic/Latina patients, especially those enrolled in Medicaid Managed Care (MMC), experiencing the longest delays. Qualitative findings converged with these patterns by identifying potential experiences and barriers described alongside delays, including symptom dismissal among younger and postpartum patients, limited understanding of diagnosis and treatment, insurance-related barriers, logistical challenges, and disconnect with clinicians who didn’t share the patients’ identity, often with patients of a different race. These qualitative experiences were consistent with some patterns observed in the quantitative analysis. Although age-specific quantitative analyses were not performed, qualitative findings expanded understanding of how younger patients may experience unique barriers during the diagnostic and treatment journey, suggesting an important area for future investigation, particularly regarding potential interactions with race and ethnicity.

#### 3.3.2 Divergence

Areas of divergence highlight limitations of claims-based measures. Some may more appropriately reflect differences in the stage of care being examined rather than conflicting findings across datasets. Quantitative analyses primarily evaluated healthcare utilization and treatment initiation following diagnosis, whereas qualitative narratives frequently emphasized experiences occurring before diagnosis, including symptom interpretation, help-seeking, diagnostic uncertainty, and communication experiences. These findings therefore represent complementary perspectives on different phases of the care continuum and identify important areas for future investigation.

Although rurality did not confer treatment delays quantitatively, qualitative narratives described isolation, fragmented coordination, and lack of access to supportive services in rural settings. Similarly, higher mammography rates among Black patients in claims data contrasted with qualitative accounts of delayed escalation and misattributed symptoms. These divergences suggest that utilization of metrics alone may not fully capture the appropriateness and timing of diagnostic evaluation.

#### 3.3.3 Expansion

Qualitative findings expanded understanding beyond what could be measured in administrative claims data by identifying emotional, relational, and practical dimensions of care experiences. These experiences may be relevant to adherence, symptom reporting, and treatment intensity; factors that extend beyond administrative measures of timeliness.

**Table 5** offers a joint display of the integrated mixed-methods findings, using the qualitative themes as the anchor and positioning the quantitative results as contextual signals. This structure highlights key areas of convergence, divergence, and expansion across the datasets, offering a comprehensive picture of the multi-level factors shaping TNBC care and providing a foundation for future research:

**Table 5:** Mixed-methods joint display demonstrating convergence and divergence: qualitative themes anchored with related quantitative findings.

Qualitative theme	Related quantitative patterns	Convergence: areas of overlap	Divergence: areas of difference	Expansion: new insights from qualitative data
Theme 1: Experiences of dismissal and delayed recognition	Significant delays in time to neoadjuvant treatment in MMC among patients of color compared to White patients: • 63.2 days (Hispanic/Latina) • 82.1 days (Black) • vs 44.2 days (White) Lower utilization of advanced diagnostic services: • Commercial digital tomosynthesis: o 56% (Hispanic/Latina) o vs. 68% (White) • MMC digital tomosynthesis: o 45% (Hispanic/Latina) o 55% (Black) o vs. 62% (White) • MMC diagnostic mammography: o 80% (Hispanic/Latina) o vs. 86% (White)	Patterns of under-triage and delayed escalation described qualitatively parallel quantitative patterns showing longer diagnostic-to-treatment intervals in specific race/ethnicity and insurance groups.	Quantitative data show screening use, but cannot detect minimization of postpartum symptoms or “too young” messaging.	Symptom misattribution and age-based assumptions shape the diagnostic experience, factors not measurable in claims.
Theme 2: Diagnosis- and care-related communication gaps and information overload	Lower utilization of diagnostic and informational services among Hispanic/Latina patients compared with White patients: • Genetic counseling: 8% vs. 14% (Commercial; p=0.0006) • Digital tomosynthesis: 56% vs. 68% (Commercial; p=0.0001) • Diagnostic mammography: 80% vs. 86% (MMC; p=0.0108)	The communication challenges described by patients resonate with observed variation in early-phase timing, where clarity of the diagnostic pathway is critical. These may help contextualize quantitative differences in treatment initiation timing by influencing patients’ understanding of and navigation through the care pathway.	Timing metrics cannot reflect distress, confusion, or cognitive overload at diagnosis.	Emotional overwhelm and the need for clearer communication are important contextual factors beyond what quantitative data capture.
Theme 3: Feeling unseen: identity, representation, and trust in care	Significant delays in time to neoadjuvant treatment in MMC among patients of color compared to White patients: • 63.2 days (Hispanic/Latina) • 82.1 days (Black) • vs 44.2 days (White)	Qualitative descriptions of cultural mismatch, limited representation and mistrust align with quantitative inequities concentrated in early pathways for Black and Latina women.	Quantitative models do not measure trust, cultural resonance, or interpersonal dynamics.	Data show relational and identity-based drivers of disparities that are invisible to claims-based analyses.
Theme 4: Administrative systems as obstacles to care navigation	Prolonged overall adjusted time to neoadjuvant treatment: • 33.9 days (commercial) • 61.2 days (MMC)	The administrative hurdles described qualitatively parallel longer adjusted times in MMC populations.	Not all insurance friction appears as a measurable delay; some administrative burden does not translate to timing differences.	Data reveals emotional, financial, and logistical strain related to insurance interactions; absent from quantitative datasets.
Theme 5: Fragmented care and poor coordination	Lower utilization of selected diagnostic services among rural patients compared with non-rural patients in the commercial population: • Digital tomosynthesis: 56% vs. 66% (p=0.0001) • Diagnostic MRI: 27% vs. 34% (p=0.0356) • Telemedicine: 18% vs. 26% (p=0.0034) No significant differences in adjusted time to neoadjuvant treatment initiation by rurality: • Commercial: Rural 36.0 days vs. Urban 34.6 days • MMC: Rural 54.4 days vs. Urban 68.9 day	Coordination challenges described by rural participants coexist with quantitative patterns showing no delay in treatment initiation.	Quantitative “no delay” contrasts with qualitative accounts of limited options, isolation, and self-coordination burden	Data highlights access gaps (trials, supportive services) that do not manifest as delays but still affect care quality.
Theme 6: Challenges in interpreting symptoms and navigating unfamiliar medical information	Lower utilization of diagnostic and informational services among Hispanic/Latina patients compared with White patients: • Genetic counseling: 8% vs. 14% (Commercial; p=0.0006) • Digital tomosynthesis: 56% vs. 68% (Commercial; p=0.0001) • Diagnostic mammography: 80% vs. 86% (MMC; p=0.0108)	Qualitative accounts of confusion and unclear plans may be connected with the underuse of certain diagnostic services	Quantitative patterns do not measure appropriateness of modality choice or adequacy of explanation	Data reveals how understanding and navigation shape engagement; elements not present in claims
Theme 7: Living with uncertainty, distress, and emotional burden	Differences in healthcare engagement and support-related services: • Oncology follow-up visits: 9% vs. 19% (Hispanic/Latina vs. White; Commercial; p=0.0001) • Oncology follow-up visits: 15% vs. 19% (Black vs. White; Commercial; p=0.0100) • Telemedicine utilization: 32% vs. 24% (Hispanic/Latina vs. White; Commercial; p=0.0006) • Telemedicine utilization: 43% vs. 34% (Hispanic/Latina vs. White; MMC; p=0.0079)	Emotional narratives offer context for why early-phase disparities may be more pronounced even when later timing is similar.	Quantitative data does not capture psychological strain or fear that persists throughout care.	Highlights mental health needs and anticipatory guidance gaps; key intervention targets beyond timing metrics.
Theme 8: Disruptions to daily life, roles, and responsibilities	Lower utilization of services among rural patients despite no treatment-initiation delays: • Digital tomosynthesis: 56% vs. 66% (Rural vs. Non-rural; Commercial; p=0.0001) • Diagnostic MRI: 27% vs. 34% (Rural vs. Non-rural; Commercial; p=0.0356) • Telemedicine: 18% vs. 26% (Rural vs. Non-rural; Commercial; p=0.0034)	Practical barriers parallel patterns of delay among MMC enrollees and certain race/ethnicity groups	Quantitative data show no rural delay despite strong qualitative evidence of geographic burden.	Reveals day-to-day constraints (childcare, hourly work, travel) critical to understanding access barriers.
Theme 9: Unexpected treatment experiences and feeling underprepared	No quantitative measures directly captured treatment preparedness, anticipatory guidance, or perceived readiness for treatment.	Qualitative accounts contextualize why timely initiation may not equate to preparedness or sustained engagement.	Timing measures treat all “on-time” starts as equal, though preparedness varies dramatically.	Identifies anticipatory guidance gaps that influence adherence, symptom reporting, and ED use.

Collectively, the integrated findings suggest that disparities observed in treatment initiation timing are unlikely to reflect a single barrier or isolated event. Rather, quantitative differences appear to occur within a broader context of interpersonal, administrative, informational, and practical challenges described by participants. While claims data identified where differences occurred, qualitative findings provided insight into how patients experienced and navigated those challenges across the care continuum.

## 4 Discussion

### 4.1 Summary of main findings

This mixed-methods study integrated claims data from 6,345 patients with TNBC and qualitative interviews with 42 patients to examine treatment timeliness, healthcare utilization, and patient experiences across the care continuum. Quantitative findings identified differences in treatment initiation timing, healthcare utilization, and diagnostic service utilization across insurance and racial/ethnic groups. Qualitative findings provided context for these patterns by highlighting experiences of delayed symptom recognition, communication challenges, administrative barriers, fragmented care, identity-related concerns, and substantial emotional and practical burdens associated with TNBC care.

Areas of convergence across datasets were particularly evident in early phases of the care pathway. Quantitative analyses demonstrated longer adjusted times to neoadjuvant treatment initiation among Black and Hispanic/Latina patients, particularly within MMC populations, while qualitative findings described experiences of dismissal, delayed recognition, communication challenges, insurance-related barriers, and difficulties navigating care. Although these findings cannot establish causal relationships, together they suggest that disparities in treatment initiation occur within a broader context of interpersonal, structural, and informational challenges.

Areas initially characterized as divergence were more appropriately interpreted as reflecting different phases of the care continuum rather than conflicting findings. Quantitative analyses primarily captured healthcare utilization and treatment initiation following diagnosis, whereas qualitative narratives frequently focused on experiences occurring before diagnosis, including symptom appraisal, delayed help-seeking, diagnostic uncertainty, and challenges obtaining appropriate evaluation. Similarly, although rural patients did not consistently experience longer adjusted treatment initiation times, they described fragmented care, limited specialty access, geographic isolation, and greater self-coordination burden. These findings suggest that administrative measures of timeliness alone may not fully capture patient experiences of access and care quality.

Qualitative findings also expanded understanding beyond what could be measured in claims data. Participants described emotional overwhelm at diagnosis, uncertainty throughout treatment and survivorship, feeling unseen or misunderstood within healthcare interactions, disruptions to work and caregiving responsibilities, and feeling underprepared for treatment-related consequences. These experiences highlight dimensions of cancer care that influence quality of life and care experiences but are largely invisible within administrative datasets.

Insurance type emerged as an important contextual factor across both data sources. Medicaid beneficiaries frequently described administrative complexity, coverage barriers, and delays associated with insurance processes, while commercially insured patients also reported financial strain, work-related constraints, and challenges navigating care. Together, these findings suggest that barriers to timely, coordinated TNBC care extend beyond insurance coverage alone and involve broader structural and practical challenges.

Collectively, the integrated findings suggest that disparities observed in treatment initiation timing are unlikely to reflect a single barrier or isolated event. Rather, quantitative differences appear to occur within a broader context of interpersonal, administrative, informational, and practical challenges described by participants. While claims data identified where differences occurred, qualitative findings provided insight into how patients experienced and navigated those challenges across the TNBC care continuum. These findings underscore the value of integrating patient experience data with administrative measures to inform more equitable, patient-centered approaches to TNBC care delivery.

### 4.2 Clinical practice and implementation considerations

The convergence of structural, communication, and experiential barriers identified in this study suggests opportunities to strengthen TNBC care delivery across several points in the care continuum. Role-based navigation models may help address care fragmentation by positioning trained lay or non-clinical navigators as a primary point of contact for patients, with structured escalation pathways to oncology nurses, social workers, financial counselors, and other clinical team members. Navigation activities may include biopsychosocial needs screening, practical support for transportation and scheduling, language-concordant education, treatment literacy support, survivorship planning, and linkage to financial or symptom-management resources.

Findings also suggest the potential value of strengthening early detection and diagnostic escalation pathways, particularly for symptomatic patients under age 40 or those with persistent or unexplained breast symptoms. Standardized approaches to expedited imaging, symptom-based awareness, and timely follow-up may reduce opportunities for delayed diagnosis. Communication and health literacy practices may be strengthened through plain-language care plans, bilingual materials, visual aids, teach-back methods, and structured pretreatment education. When paired with language access and navigation support, telehealth-based education may also reduce geographic, transportation, and work-related barriers.

Reducing administrative and financial burden may require earlier integration of financial navigation, social work support, oncology-specific prior authorization processes, and flexible scheduling models. Efforts to enhance trust and cultural responsiveness may include person-centered communication training, oncology-specific implicit bias training, and increased access to race- and language-concordant clinicians, navigators, and peer support. Supportive care and survivorship services, including psycho-oncology, symptom management, nutrition, integrative care, peer mentoring, and early palliative care, may further address emotional burden and unmet needs across treatment and survivorship.

**Figure 3:**
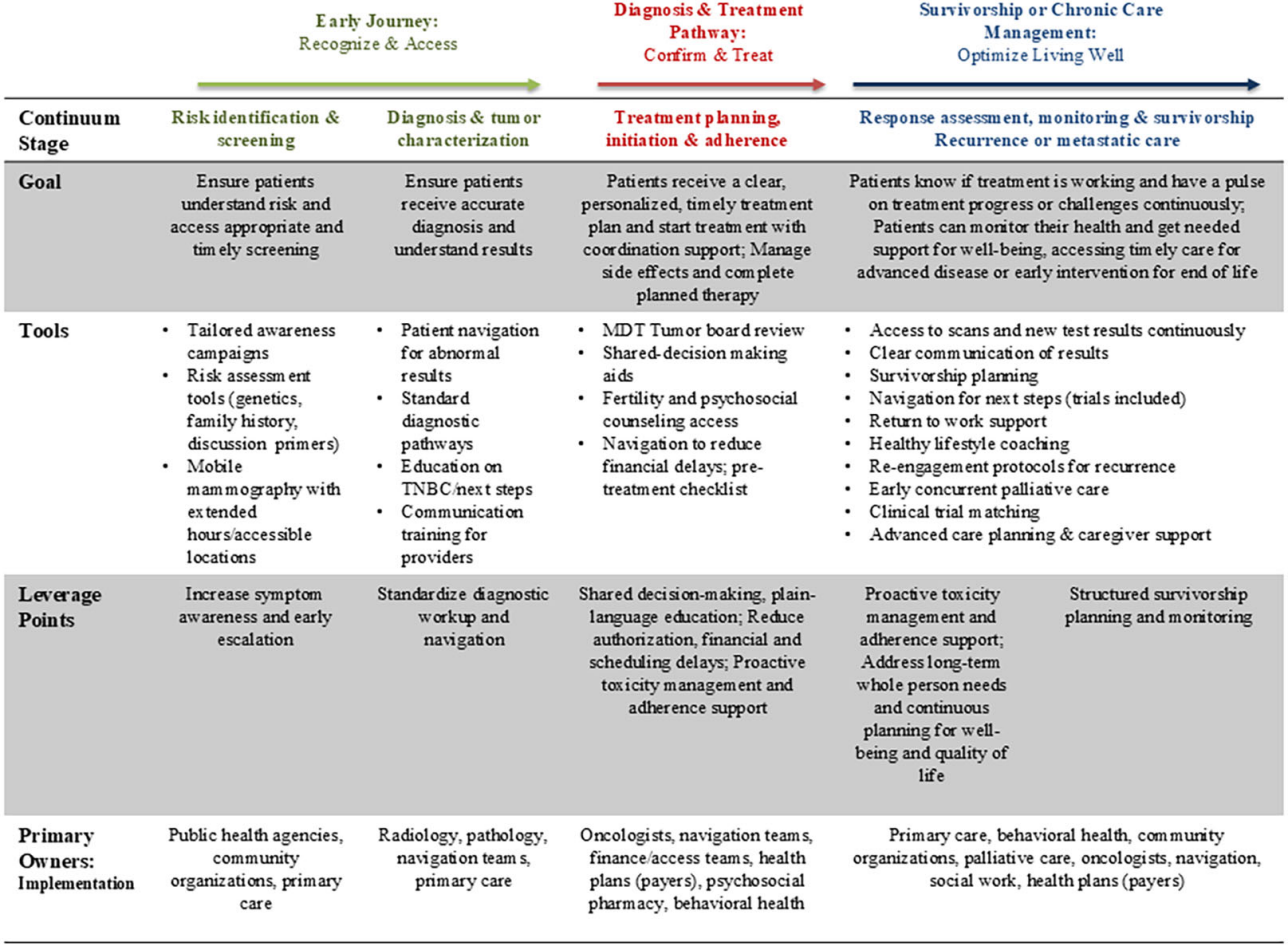
Proposed health system–anchored TNBC care continuum: priority leverage points for intervention, tools, and ownership. Author-developed conceptual TNBC care continuum informed by study findings and existing cancer care delivery frameworks; presented as a hypothesis-generating model to guide future research and intervention development.

Overall, these findings support a proposed health system–anchored TNBC care continuum that identifies priority intervention points from symptom recognition and diagnosis through treatment initiation, survivorship, and recurrence management. **Figure 3** presents a hypothesis-generating conceptual framework illustrating intervention opportunities, implementation strategies, and key stakeholders who may support more coordinated, patient-centered TNBC care.

### 4.3 Limitations

The qualitative findings rely on patient self-report, which depends on participants’ ability to accurately recall symptoms, diagnostic timelines, and treatment histories; accounts may be incomplete, imprecise, or shaped by emotional memory, introducing potential recall bias. Because participants were recruited through an existing patient panel using purposive recruitment targets, the sample may not fully represent the diversity of experiences among all individuals with TNBC. Individuals participating in patient panels may differ from the broader TNBC population in ways that could influence reported experiences and findings may therefore not generalize to individuals with different cultural backgrounds, disease courses, or care settings.

Because TNBC identification relied on a claims-based proxy algorithm rather than direct receptor-status information, and because this algorithm was not validated within the present study dataset, some degree of subtype misclassification may have occurred. Delayed, omitted, or incompletely captured receptor-targeted therapies may have resulted in the inclusion of patients with non-TNBC breast cancer subtypes, potentially biasing estimates either toward or away from observed differences. Emerging validation work evaluating treatment-based proxy approaches for TNBC has suggested strong predictive performance for HER2-negative disease but lower predictive performance for HR-negative disease, highlighting the potential for subtype misclassification when treatment patterns are used to infer biologic status (26). Because sensitivity analyses using alternative TNBC identification approaches were not conducted, the magnitude and direction of any potential bias remain uncertain. Additionally, TNBC-specific ICD-10-CM coding became available after the study period and, therefore, was not available for incorporation into the present analysis.

Treatment cohort assignment relied on claims-based operational definitions anchored to the timing of systemic therapy relative to surgery. Although this approach enabled categorization of treatment patterns within administrative data, no universally accepted timing threshold exists for cohort assignment in the literature, and studies have used varying windows for treatment classification. Consequently, some degree of treatment cohort misclassification may have occurred, particularly among patients whose treatment timing occurred near classification boundaries, potentially biasing estimates either toward or away from observed differences. Because sensitivity analyses using alternative treatment timing thresholds were not conducted, the magnitude and direction of any potential effect remain uncertain. Model diagnostics, interaction analyses, and sensitivity analyses were not conducted; therefore, potentially important subgroup relationships, model-specific effects, and the robustness of findings to alternative analytic assumptions may not have been fully evaluated. Administrative claims data lack clinical detail, including cancer subtypes, stage at diagnosis, symptom onset, or markers of communication quality and emotional experience elements central to understanding patients’ diagnostic and treatment journeys. Mortality data were unavailable, and the most recent claims were from 2021, limiting insight into more contemporary care patterns. Because the claims data largely predated approval and routine adoption of pembrolizumab for early-stage TNBC, the findings may not fully reflect current immunotherapy-era treatment patterns.

The dataset included only a subset of commercial and MMC beneficiaries in the MORE2 Registry, excluding fee-for-service enrollees, and therefore may not reflect the full TNBC population. Race and ethnicity fields were missing for 21% of the Medicaid cohort and 85% of the commercial cohort and were imputed using Acxiom near-neighborhood estimates linked at the 9-digit ZIP Code level. Although distributions after imputation were generally consistent with enrollment data and publicly available estimates, the high proportion of imputed race and ethnicity data, particularly in the commercial cohort, limits the precision of race- and ethnicity-stratified estimates; neighborhood-based proxy approaches may not fully capture individual racial or ethnic identity, multiracial identity, cultural affiliation, or intersectional experiences. Use of aggregate geographic characteristics to infer individual-level attributes may also introduce ecological bias, whereby characteristics of a surrounding population do not accurately reflect an individual member (27). Prior work suggests that more granular geographic units may improve precision compared with broader geographic measures, although residual bias may remain (23, 27). Therefore, race- and ethnicity-stratified findings should be interpreted as exploratory and hypothesis-generating rather than definitive estimates of individual-level racial or ethnic differences.

Additionally, rural–urban differences observed in claims may underestimate earlier delays, as claims capture only diagnosed encounters, not pre-diagnostic barriers. There was also an overrepresentation of non-rural versus rural TNBC patients in the datasets. The analysis did not incorporate measures of pandemic intensity or service disruptions, which may have influenced care patterns during 2020–2021, nor did it include radiation therapy data, which may partially explain some delays in adjuvant treatment.

### 4.4 Conclusion

This study offers a novel contribution to the body of literature in the following ways:

1. While much of the existing TNBC quantitative research is epidemiologic in nature and focused on treatment patterns and survival outcomes, and the qualitative literature predominantly describes disparities after diagnosis for high-risk populations, this study illuminates early-phase experiences in the TNBC care pathway, before and leading to diagnosis, from symptom onset, to help-seeking, through diagnostic work-up, into treatment decision-making and initiation of treatment.
2. This research explores patient-perceived factors related to timely, high-quality care, and offers hypothesis-generating insight into interpersonal and system-level challenges that may shape the early TNBC care pathway.
3. To our knowledge, there are few intersectional frameworks (28) exploring patient experiences with TNBC care in the early part of the pathway from an intersectional lens, exploring race or ethnicity, age, insurance type, language, and geography.

This study highlights how clinical, structural, communicative, and psychosocial challenges may shape access to high-quality TNBC care and patient experience, particularly for younger patients, Black and Hispanic/Latina patients, Medicaid beneficiaries, and rural residents. While claims data identified observed patterns in diagnostic modality use, timeliness, and healthcare utilization, qualitative narratives provided context for how patients experienced the care pathway in relation to these patterns. These findings suggest potential opportunities for targeted interventions, including patient navigation, improved communication practices, streamlined insurance processes, culturally responsive care, and enhanced supportive and survivorship services, to support more consistent, patient-centered TNBC care.

## Data Availability

Requests regarding data availability will be considered on a case-by-case basis subject to applicable data use agreements, ethical considerations, and privacy restrictions. Requests to access the datasets should be directed to LH, (laura.housman@avalerehealth.com).
